# Supplementary material for: SyNDI: synchronous network data integration framework
Source: BMC Bioinformatics. 2018 Nov 6;19:403. doi: 10.1186/s12859-018-2426-5 (PMC6219086; doi:10.1186/s12859-018-2426-5)
Supplement: Supplementary file 5 — Files of the example presented in Additional file 4. This additional file is a zip package that contains all files used in Additional file 4. Detailed descriptions of each of these files are presented in a README file included in this zip package. (GZ 30411 kb) [file 12859_2018_2426_MOESM5_ESM.gz › SyNDITools/Meme2Fimo/meme_4.9.0_4.tar.gz!/meme_4.9.0/doc/glam2_tut.html]

glam2 Tutorial


# glam2 Tutorial

## What it does

You give glam2 a set of sequences, and it finds the strongest
motif shared by these sequences. More exactly, glam2 gives you an
alignment of segments of the sequences. Each sequence contributes at
most one segment to the alignment. glam2 assigns scores to alignments:
the score favours alignment of similar residues, and disfavours
insertions and deletions, but less so if they repeatedly occur at the
same, presumably fragile, positions. glam2 attempts to find a
maximal-scoring alignment for your sequences.

## Before starting: beware of confounding similarities

Biological sequences often have strong similarities that are not
interesting. These strong similarities need to be removed before weaker
motifs can be found. Here are some common cases:

- **Highly similar sequences**: If your set
  includes highly similar sequences (e.g. equivalent sequences from human
  and chimpanzee), you can get a subset of non-highly-similar sequences
  using purge.
- **Repeats**: Biological sequences harbour a
  complex menagerie of repetitive and simple sequence elements. These are
  often prominent motifs. If you wish to avoid them, mask them before
  using glam2. These tools mask various kinds of repeats: Repeatmasker, Censor, seg / pseg / nseg, DUST,
  XNU, DSR, GBA, SIMPLE,
  CARD, CAST, SAPS, TRF. Another approach is
  to run glam2 once, then use glam2mask
  to mask the motif found by glam2, and then run glam2 again to find
  weaker motifs.

## How it works

To use glam2 effectively, you need to understand roughly how it
works. glam2 starts from a random alignment, and makes many small,
random changes to it, which are designed to find high-scoring alignments
in the long run. The longer you let it run, the more likely it is to
find a maximal-scoring alignment.

To check that a reproducible, high-scoring motif has been found,
the whole procedure is run several (e.g. 10) times from different
starting alignments. If all runs produce identical alignments, we have
maximum confidence that this is the optimal motif. (To gain even more
confidence, consider varying the initial motif width: see below.) If a
few of the runs produce different, lower-scoring motifs, we still have
high confidence. If all the runs produce completely different
alignments, we have low confidence, and the run-length needs to be
increased.

An alternative is to check that similar, but not necessarily
identical, alignments are found repeatedly. This suggests that the
optimal motif has been found, if not the exactly optimal alignment. With
large numbers of sequences, there are so many possible alignments that
it is not feasible to find the precisely optimal one, and this is the
best that can be hoped for. Furthermore, the precisely optimal alignment
is not very meaningful: it is rather like writing a moderately accurate
value to twelve decimal places.

## Basic usage

Running glam2 without any arguments gives a usage message:

```
Usage: glam2 [options] alphabet my_seqs.fa
Main alphabets: p = proteins, n = nucleotides
Main options (default settings):
-h: show all options and their default settings
-o: output file (stdout)
-r: number of alignment runs (10)
-n: end each run after this many iterations without improvement (10000)
-2: examine both strands - forward and reverse complement
-z: minimum number of sequences in the alignment (2)
-a: minimum number of aligned columns (2)
-b: maximum number of aligned columns (50)
-w: initial number of aligned columns (20)
```

The main input to glam2 is a file of sequences in FASTA format:

```
>MyFirstSequence
GHYWVVCTGGGACH
>My2ndSequence
LLIGGPWVWWADDDF
(etc.)
```

You need to tell glam2 which alphabet to use:

```
glam2 p my_prots.fa
```

```
glam2 n my_nucs.fa
```

Use -o to write the output to a file rather than to the screen:

```
glam2 -o my_prots.glam2 p my_prots.fa
```

## Output format

The output begins with some general information:

```
GLAM2: Gapped Local Alignment of Motifs
Version 9999

glam2 p my_prots.fa
Sequences: 4
Greatest sequence length: 14
Residue counts: A=3 C=2 D=3 E=3 F=2 G=4 H=4 I=4 K=0 L=5 M=1 N=3 P=4 Q=0 R=2 S=3
T=1 V=2 W=0 Y=2 x=0
```

This is followed by several alignments, sorted in order of score.
The topmost alignment is the interesting one: the purpose of the others
is to indicate the reproducibility of the topmost one. An alignment
begins with a summary line:

```
Score: 18.0451  Columns: 6  Sequences: 4
```

Followed by the alignment itself:

```
                **..****
seq1         10 HP..D.IG 14 + 8.72
seq2          5 HPGADLIG 12 + 7.39
seq3          7 HP..ELIG 12 + 15.7
seq4          5 HP..ELLA 10 + 9.71
```

The stars indicate the *key positions* of the motif, i.e.
the conserved and presumably functional positions. Positions without
stars hold residues that are inserted between key positions. No attempt
is made to align inserted residues with each other: their column
placement is arbitrary. Gaps in key positions indicate deletions. The
plus signs indicate the strand (only meaningful when considering both
strands of nucleotide sequences with the -2 option). The rightmost
numbers are the *marginal scores* of each aligned segment, i.e.
the amount by which the total alignment score would decrease if the
segment were removed. The marginal scores do not in general sum to the
total alignment score.

The alignment is followed by a *multilevel consensus
sequence*, indicating the dominant residue types in each key position:

```
                HP  DLIG
                    E LA
```

The residue types are ordered by frequency from top to bottom,
and only those with a frequency of at least 20% are written. This
representation is borrowed from MEME.
This is followed by a matrix of the residue and deletion counts for each
key position, and insertion counts between them, along with their
scores:

```
 A  C  D  E  F  G  H  I  K  L  M  N  P  Q  R  S  T  V  W  Y Del Ins Score
 0  0  0  0  0  0  4  0  0  0  0  0  0  0  0  0  0  0  0  0   0      7.33
                                                                  0 -0.263
 0  0  0  0  0  0  0  0  0  0  0  0  4  0  0  0  0  0  0  0   0      7.98
                                                                  2 -8.61
 0  0  2  2  0  0  0  0  0  0  0  0  0  0  0  0  0  0  0  0   0      4.55
                                                                  0 -0.263
 0  0  0  0  0  0  0  0  0  3  0  0  0  0  0  0  0  0  0  0   1     -0.642
                                                                  0 -0.263
 0  0  0  0  0  0  0  3  0  1  0  0  0  0  0  0  0  0  0  0   0      4.21
                                                                  0 -0.263
 1  0  0  0  0  3  0  0  0  0  0  0  0  0  0  0  0  0  0  0   0      4.28
```

These scores sum to the total alignment score.

## Controlling speed versus accuracy

Use -n to control how long glam2 runs for, and -r to control how
many runs it does. Quick and dirty:

```
glam2 -r 1 -n 1000 p my_prots.fa
```

Slow and thorough:

```
glam2 -n 1000000 p my_prots.fa
```

If the top alignment is completely different from all the other
alignments, there is little confidence that the best possible motif has
been found, and glam2 should be re-run with higher -n. (In our
experience, increasing -n works better than increasing -r.)

## Looking at reverse strands

Use -2 to search both strands of nucleotide sequences:

```
glam2 -2 n my_nucs.fa
```

## Minimum number of sequences in the alignment

Use -z to set the minimum number of sequences that must
participate in the alignment (if you set it to more than the number of
input sequences, then all the sequences must participate):

```
glam2 -z 10 p my_prots.fa
```

## Bounding the motif width

Use -a and -b to set lower and upper bounds on the number of key
positions in the motif:

```
glam2 -a 10 -b 20 n my_nucs.fa
```

We do not recommend setting -a equal to -b, as this dramatically
constrains glam2's flexibility.

## Setting a good starting point for the motif width

glam2 automatically adjusts the number of key positions so as to
maximize the alignment score, but it sometimes has trouble with this,
for two reasons:

1. It can only add or remove one key position at a time (unlike
   GLAM and A-GLAM). If it needs to add or remove multiple key positions
   in order to increase the score, passing through lower-scoring
   alignments on the way, it will be averse to doing this.
2. It needs to add or remove key positions in a reversible
   fashion, which becomes difficult or impossible when the number of
   sequences is large, especially if the number of sequences is much
   greater than the length of each sequence. In extreme cases, the motif
   width may never change from its initial value.

You can help glam2 by using -w to set the initial number of key
positions to a ballpark value:

```
glam2 -w 100 -a 50 -b 200 n my_nucs.fa
```

In this example, we guess that the optimal number is around 100,
and allow it to vary between 50 and 200.

## Tuning deletion and insertion preferences

Use -D and -E to tune deletion preferences, and use -I and -J to
tune insertion preferences. The relative values of -D and -E control
glam2's aversion to deletions: increasing -E relative to -D makes it
more averse. Likewise, increasing -J relative to -I makes glam2 more
averse to insertions. The absolute values of -D and -E control how much
glam2 prefers deletions to occur at the same (fragile) positions: if -D
and -E are both low, it strongly prefers deletions to occur at the same
positions, otherwise not. Likewise, if -I and -J are both low, it
strongly prefers insertions to occur at the same positions. To turn off
deletions and insertions completely, set -E and -J to huge values:

```
glam2 -E 1e99 -J 1e99 n my_nucs.fa
```

## Unusual residue abundances

If the input sequences have unusual residue abundances, glam2 may
interpret these as being a motif. If this interpretation is not
appropriate, you can fix it by changing glam2's idea of usual residue
abundances. The best way to do this is with an alphabet file. For example, you could
specify the average abundances for the taxonomic group that the
sequences come from. Alternatively, you can use -q 1 to make glam2
estimate residue abundances from the input sequences:

```
glam2 -q 1 p my_prots.fa
```

This works well if the input sequences are much larger than the
motif.

## Significance of glam2 motifs

glam2 will always find the best motif that it can, even if you
give it random sequences. Thus, it would be nice to know whether or not
a motif found by glam2 is stronger than what would be found in random
sequences. Here are two ways to answer this question:

1. **Brute force**: This method is slow but good.
   Randomly shuffle the letters in each of your sequences (for example,
   using shuffleseq from EMBOSS).
   Run glam2 on the shuffled sequences, with exactly the same options as
   for the original sequences. Record the top alignment score. Reshuffle
   and re-run glam2 many times (e.g. 100 times, or maybe 20 times). If,
   say, 99 out of 100 shuffles produce lower scores than the original
   sequences, then the motif is probably significant. You can speed this
   up by lowering -n and/or -r, as long as the same values are used for
   the original and shuffled sequences. Once significance has been
   established, you can then align the motif more accurately by increasing
   -n and/or -r.
2. **Decoy approach**: This method is faster, but
   may fail to spot significant motifs, especially when the number of
   sequences is small. Randomly shuffle the letters in each of your
   sequences, and concatenate each shuffled sequence to its parent
   sequence, separated by, say, ten wildcard letters (e.g. 'x'). Run glam2
   once on this dataset. Does the top alignment more often include parent
   segments than shuffled segments, and/or do parent segments get higher
   marginal scores than shuffled segments? If so, the motif is probably
   significant. This can be quantified with a Wilcoxon signed rank test:
   see a statistics textbook or the Web.

These methods use shuffled versions of the original sequences:
control sequences obtained in some other way may be used instead, but
should be chosen with care.
